# Monitoring the variation in the gut microbiota of captive woolly monkeys related to changes in diet during a reintroduction process

**DOI:** 10.1038/s41598-021-85990-0

**Published:** 2021-03-22

**Authors:** Camilo Quiroga-González, Luis Alberto Chica Cardenas, Mónica Ramírez, Alejandro Reyes, Camila González, Pablo R. Stevenson

**Affiliations:** 1 Laboratorio de Ecología de Bosques Tropicales y Primatología (LEBTYP), Departamento de Ciencias Biológicas, Facultad de Ciencias, Universidad de Los Andes, Bogotá, Colombia; 2 Grupo de Investigación en Biología Computacional y Ecología Microbiana (BCEM), Max Planck Tandem Research Group in Computational Biology, Universidad de Los Andes, Bogotá, Colombia; 3 The Edison Family Center for Genome Sciences and Systems Biology, Washington University School of Medicine, Saint Louis, MO USA; 4 Centro de Investigaciones en Microbiología y Parasitología Tropical (CIMPAT), Departamento de Ciencias Biológicas, Facultad de Ciencias, Universidad de Los Andes, Bogotá, Colombia

**Keywords:** Ecology, Conservation biology, Microbiology, Metagenomics

## Abstract

Microbiome is known to play an important role in the health of organisms and different factors such as diet have been associated with modifications in microbial communities. Differences in the microbiota composition of wild and captive animals has been evaluated; however, variation during a reintroduction process in primates has never been reported. Our aim was to identify changes in the bacterial composition of three individuals of reintroduced woolly monkeys (*Lagothrix lagothricha*) and the variables associated with such changes. Fecal samples were collected and the V4 region of the *16S rRNA* gene was sequenced to determine gut microbial composition and functionality. Individual samples from released individuals showed a higher microbial diversity after being released compared to before liberation, associated with changes in their diet. Beta diversity and functionality analysis showed separation of samples from released and captive conditions and the major factor of variation was the moment of liberation. This study shows that intestinal microbiota varies depending on site conditions and is mainly associated with diet diversity. The intake of food from wild origin by released primates may promote a positive effect on gut microbiota, improving health, and potentially increasing success in reintroduction processes.

## Introduction

Gut microbiota is important for animal’s physiological activities and health, influencing aspects such as the ability to extract energy and nutrients from some food components^1^. It encodes metabolic functions that many animals are not able to synthesize by themselves^2^. The highly diverse microbiome is composed by microorganisms from all different domains of life and viruses; among them, bacteria play the most important role, since many of them have roles in the protection against pathogens and degradation of organic substances facilitating the host nutrient assimilation^3,4^.

When animals are kept in captivity, there is a rising concern that it may result in an alteration of the stable structure of the microbial community and even the loss of microbial diversity that may contribute to failures of reintroduction efforts^5^. Those changes will be reflected in a reduced health status of individuals. A reduced diversity of gut microbiota can produce dysbiosis, decreasing some microbial functional groups and altering different metabolic pathways, which may compromise the host health making it more prone to acquire diseases^6,7^. Several studies have demonstrated that gut microbial diversity is affected by captive conditions^8–10^. Specifically, primates show a consistent reduction in bacterial alpha diversity compared to animals in natural conditions, losing their native microbial biodiversity^9,11^. For most primates, captive environments represent an extreme change compared to their environment in the wild, related primarily to the diet composition, but also to other aspects such as antibiotic use, stress, and variation in social interactions can lead to changes in gut bacterial communities^12–14^.

For instance, diet and phylogeny are the most strongly associated factors shaping the gut microbiota^2,11,15,16^. Dietary niche plays a major role in determining the gut microbiota structure and composition across diverse host species^2,11,17^. It has been found that herbivores have the richest microbiota at the phylum and genus level followed by omnivores and carnivores^2^. In fact, a more complex and diverse microbiota is necessary to optimally exploit the hard to digest lignin and cellulose, and to process the secondary metabolites present in leaves^18,19^. Primates in the wild eat a vast variety of plants and captivity implies a dramatic change in that diet, which may alter their gut microbiota^9,20^. Folivorous animals converge in microbial traits, nevertheless, studies have shown that host phylogeny has a stronger effect in structuring the microbial community than host dietary niche^2,21,22^; however, when animals are phylogenetically closely related, the diet has a strong influence in the microbial community^16^.

Several studies have focused on comparing differences in gut microbiome communities between captive and free ranging individuals in different species^9,10,23–26^. In these studies, microbiome alpha diversity was consistently higher in samples from wild individuals compared to captive ones, furthermore, beta diversity showed that individuals in captivity harbor different microbial communities compared to wild ones. However, to our knowledge, no studies have compared the gut microbiota during a reintroduction process in primates. From this perspective, identifying the variation of gut microbiota in reintroduction processes in primates is of great importance to infer how they are exploiting their new habitat when released to wild areas. In this study, we aimed to identify changes in gut microbiota during the rehabilitation and reintroduction of three woolly monkeys (*Lagothrix lagothricha*) in Colombia. In addition, we investigated if temporal changes in diet after individuals release are associated with microbial diversity. We predicted that microbial and functional communities grouped together by the different conditions (Captive and Released), with individuals having a higher gut microbiome diversity after released to a wild environment. This work will provide valuable information on how the gut microbiota is affected during reintroduction processes, and on the resilience and plasticity of bacterial communities under different ecological conditions.

## Results

We performed a characterization of the gut microbiota for three woolly monkeys (Hodor: Adult Male; Yara: Adult Female; Arya: Juvenile Female) during a reintroduction process, for this, we analyzed nineteen samples, nine while captive and ten after released.

**Figure 1:**
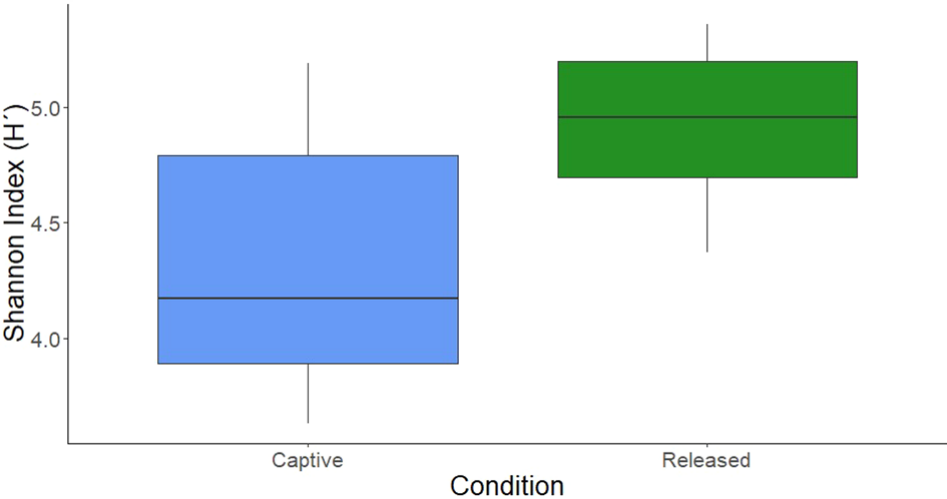
Bacterial Shannon diversity index for the different conditions of the individuals, Captive (n = 9) and Released (n = 10). Samples from the recaptured individual are considered in the captive condition in this analysis. Graphic shows significant differences between the two conditions (ANOVA, *F*_1,17_ = 9.22, *p* = 0.017).

Shannon diversity index between conditions showed that samples taken from the individuals after the liberation had a significantly higher diversity in bacterial communities when compared to captive ones (ANOVA, *F*_1,17_ = 9.22, *p* = 0.017) (Fig. 1).

When analyzing alpha diversity as a function of time, we found that Arya had a higher bacterial Shannon diversity compared to the other two individuals while in captivity. Shannon diversity start to increase at the moment of the liberation for Hodor and Yara, but Arya appeared not to change. Shannon bacterial diversity start to decrease around 50 days after liberation. Samples collected from the recaptured individual showed the same tendency, where Shannon diversity started to increase at the moment of liberation but decreased again when the individual returned to captivity. Chao bacterial diversity showed the same trend where Arya continued to have a higher diversity compared to the other two individuals and in average bacterial diversity increased in two of the three animals after the liberation (Fig. S1).

When identifying variables associated to the variation in bacterial composition, we found that the percentage of time that individuals engaged in social interactions was higher in captivity compared to the time after being released (Table S2). As for the time after the liberation, we found that “days after release” was not associated with bacterial diversity for the two indices evaluated (Fig. S2). When we performed a model selection criterion (AIC)(Table S1), we found that the percentage of food the individuals were feeding from the forest had a positive effect and was the variable that best explained bacterial diversity for Shannon (*p* = 0.023, R^2^ = 0.42, β = 0.008) and marginally for Chao 1 (*p* = 0.069, R^2^ = 0.77, β = 0.776) index (Fig. S2).

We found that Shannon diet diversity was significantly different between captive and released conditions (ANOVA, *F*_1,17_ = 14.86, *p* < 0.001) being higher in released (Fig. S3a). We also found that the diet diversity seems not to change during the time the individuals were in captivity and started to increase after liberation and rapidly stabilized. When the adult female was recaptured and returned to captivity, diet diversity decreased (Fig. S3b). Additionally, we found that the released individuals shows a shift on diet where they start consuming more fruits and vegetables compared to when they were in captivity (Fig. S4).

**Figure 2:**
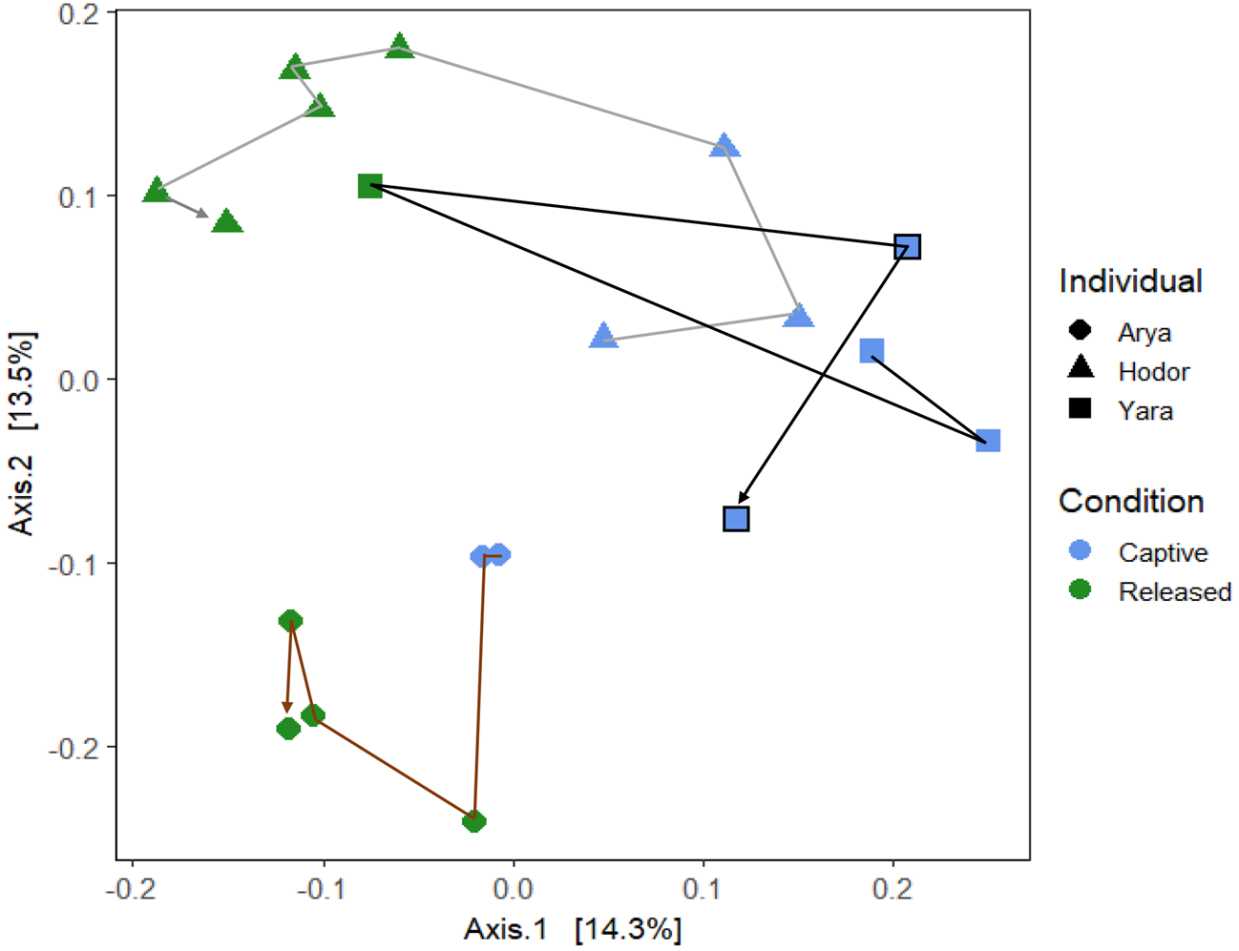
Unweighted UniFrac distances of *16S rRNA* gene amplicons data grouping by condition and name of the individuals. Samples with a black border represent samples from an individual upon return to captivity after one month in the wild. Arrows follow the trajectory of samples from each individual over time.

Our beta-diversity analysis using a weighted-Unifrac distance showed that condition was the only variable that was associated with bacterial communities (Fig. S5; F = 4.123, R^2^ = 0.20, *p* = 0.011). These results did not change even when we included sex, age, and individual in the same model; neither *p*-value was significant for these categories, nor R^2^ was higher than 0.05. When we performed the Unweighted-Unifrac distance analysis, we found that condition and individual were the variables explaining the differences in our Adonis model (Fig. 2; F = 5.1, R^2^ = 0.33, *p* = 0.001), and individual on its own was the single variable that best explained bacterial communities (R^2^ = 0.23).

Regardless of the study site condition, the samples grouped by individual, suggesting that each individual harbors a unique bacterial community (Fig. 2). When the individuals were released, their bacterial communities changes but maintained the differences among them. Based on the PCoA plot, the largest variation among consecutive time points was observed between the samples right before and after the liberation. Interestingly, we observed that samples of the recaptured individual grouped with samples from the same individual previous to liberation, rather than with the samples from the individual after liberation.

**Figure 3:**
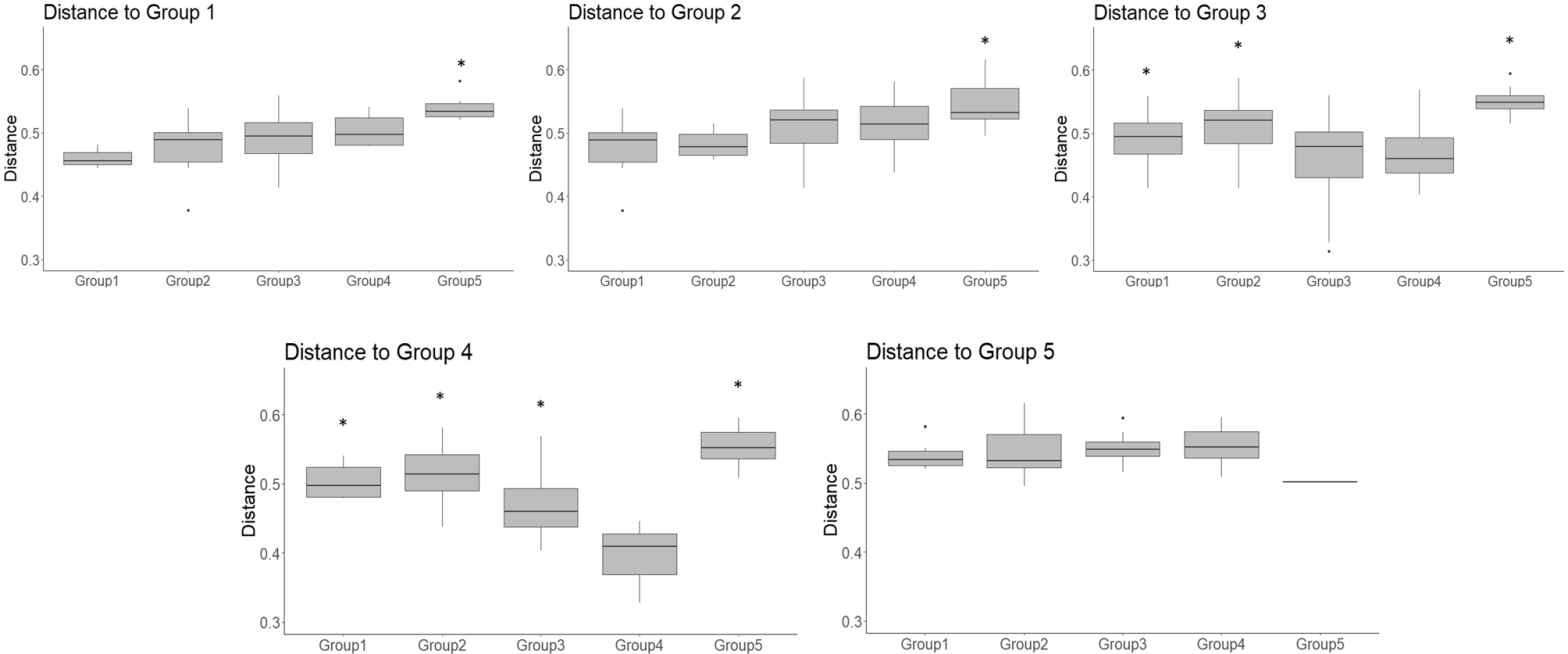
Beta group significance analysis of Unweighted distances between five categories based on the time period during the reintroduction process. Group (1) Between − 100 and − 52 days prior to liberation, Group (2) Between − 52 and 0 days prior to liberation, Group (3) Between 0 and 100 after the liberation, Group (4) From 100 days until the end of the study for the released individuals and Group (5) From 100 days until the end of the study for the recaptured individual. Asterisk represent statistical differences (*p* < 0.05) between the different groups and the group being compared.

Beta diversity group distance analysis confirmed the observations that the average distance of samples, taken at closer time points, are more similar to each other than samples taken further apart. When we compared weighted distances dividing time in five group categories (see “Methods”), we only found differences when comparing groups before and after the liberation (Group 2 vs. Group 4 and Group 2 vs. Group 3), suggesting that samples taken in captivity were certainly different from the released ones. In all cases, Group 1 and Group 2 had very similar distances supporting the idea that in captivity, individuals have their own differential microbiota (Fig. S6). With the unweighted distances we obtained similar results, showing that distances between Group 3 and Group 4 (released samples) were different from captive ones, also Group 4 was different to all other categories supporting the idea that time after liberation has an effect on restructuring the gut microbiota (Fig. 3).

**Figure 4:**
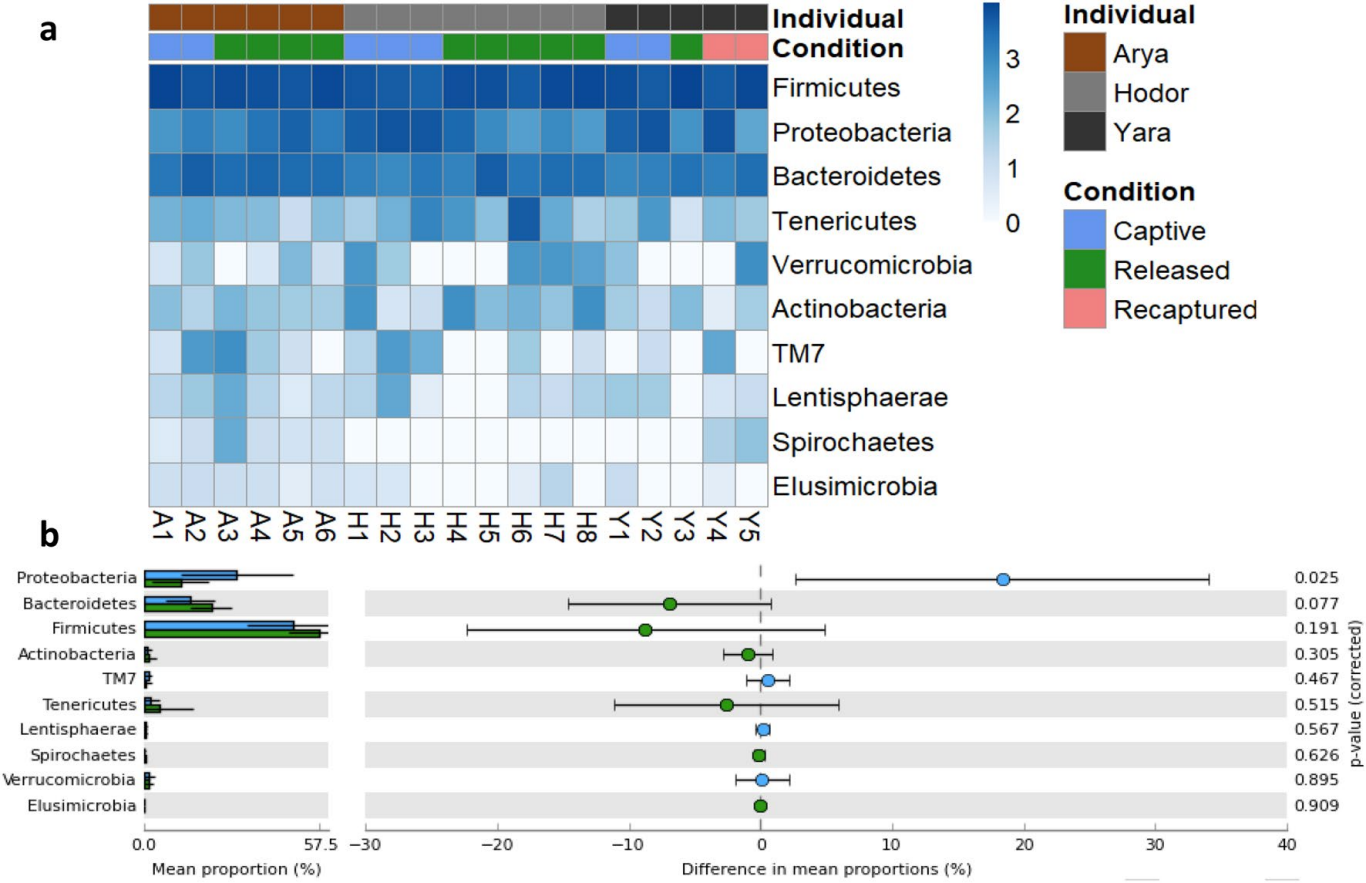
(**a**) Heatmap showing the logarithm of the abundance of bacterial phylum found in fecal samples from different conditions. Samples were sorted by individual, and within individual by time. Colored boxes on top of the heatmap indicate the condition when the sample was taken and are grouped by individual. (**b**) Extended error bar plot with a 95% confidence interval organized by the lowest *p*-value showing the differences in mean proportions between captive and released individuals and its associated corrected *p*-value (Welch’s t-test), notice that only proteobacteria had a significant deviation in the mean proportion.

Given the change in bacterial communities between conditions, we evaluated bacterial composition to determine which phyla and genera were responsible for this variation. We found that Firmicutes (53.3%), Proteobacteria (20.6%), and Bacteroidetes (18.7%) were the phyla representing the higher abundance in all the analyzed samples (Fig. 4a). All phyla found in this study were present in both conditions, but Proteobacteria was the only one with a significant variation, being higher in the captive condition in two of the three study subjects (Fig. 4b). Samples from the recaptured individual seemed to have the same tendency, diminishing Proteobacteria abundance after liberation, and increasing when sampled back in captivity. T-Test of Bacteroidetes and Firmicutes showed a tendency of higher abundance in released individuals, but no statistical significance was found (*p* = 0.077 and *p* = 0.191 respectively).

At the genus taxonomic level, we found that 17 genera were different with the *p*-value we choose to reduce the dimension of the heatmap (*p* < 0.1) (Fig. S7a). The most abundant genus was *Faecalibacterium* followed by *Ruminobacter*. Interestingly, three bacterial genera (*Campylobacter*, *Acinetobacter*, and *Lachnospira*) were present in the captive condition but completely absent in released individuals. When we performed the analysis with a *p* < 0.05 we found that only eight genera of bacteria were different, being *Bacteroides*, *Sutterella,* Unclassified Coriobacteriaceae and *Succinatimonas* higher in released individuals, and *Ruminobacter*, *Campylobacter,* Unclassified S24_7 and *Sharpea,* higher in samples from captive individuals (Fig. S7b). From the bacteria showing differences among conditions, we highlight three genera that share an interesting pattern: *Campylobacter*, is present in all samples from captivity and recapture, but completely absent in released individuals samples; *Sutterela* and *Bacteroides* have the opposite pattern, being consistently higher in samples collected after the liberation compared to when individuals were in captivity (including the recaptured individual).

**Figure 5:**
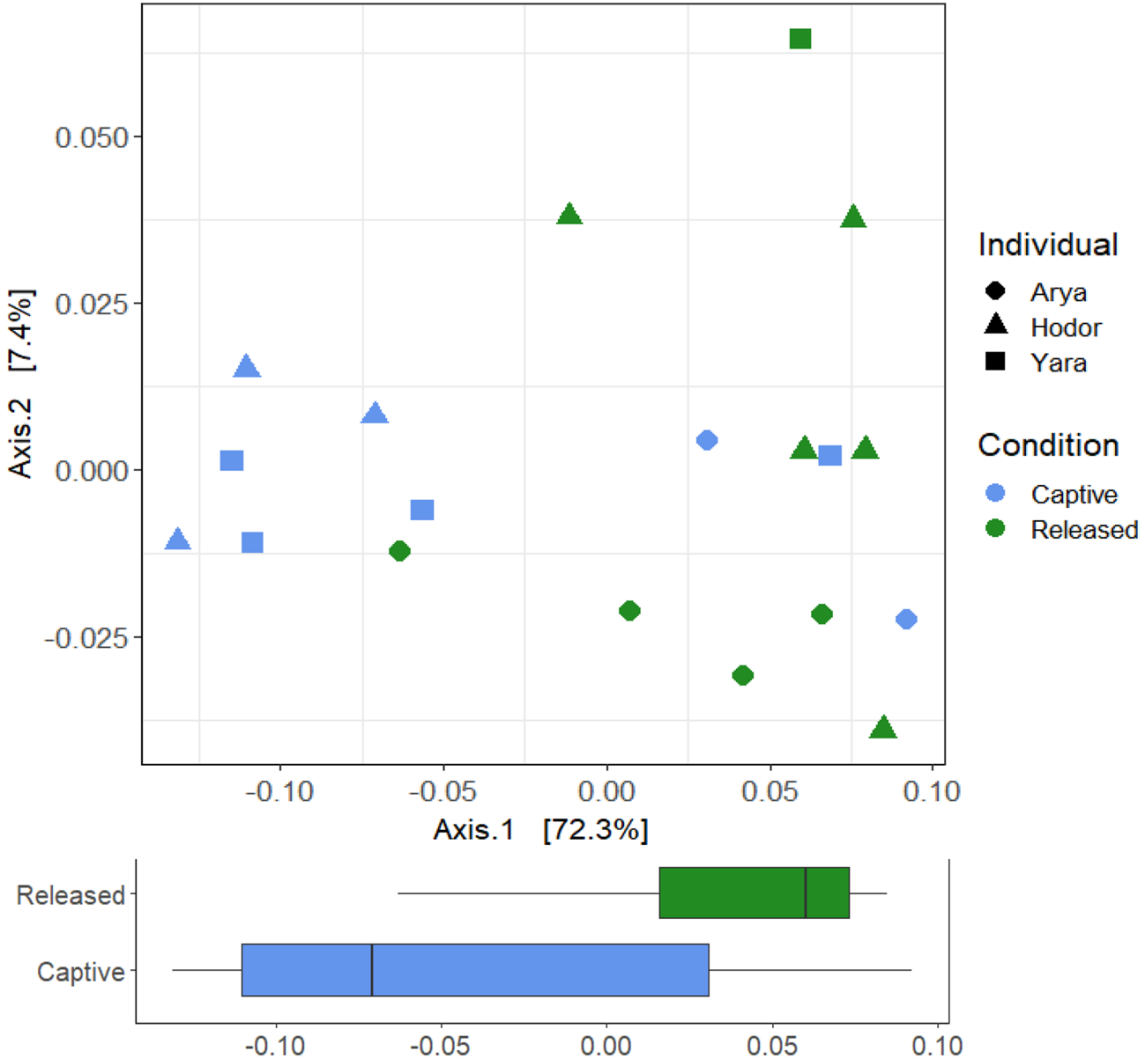
PCoA of bray–curtis distances of PICRUSt functional diversity of *16S rRNA* gene amplicons of bacterial communities in primate feces grouped by individual and condition. Samples from recaptured individual are considered as captive in this analysis. Boxplot shows the data dispersion over the first axis of the PCA between samples taken from captive and released indiciduals.

To further analyze whether the difference in bacterial composition might reveal a variation in the functional profile of the microbiota, we performed a Bray–Curtis distance analysis with the PICRUSt2 output. We found significant differences among conditions (F = 5.91, R^2^ = 0.37, *p* = 0.001) (Fig. 5). PCoA analysis showed that the main axis of variation, explaining 72.3% of the variance, showed a separation mainly due to condition.

**Figure 6:**
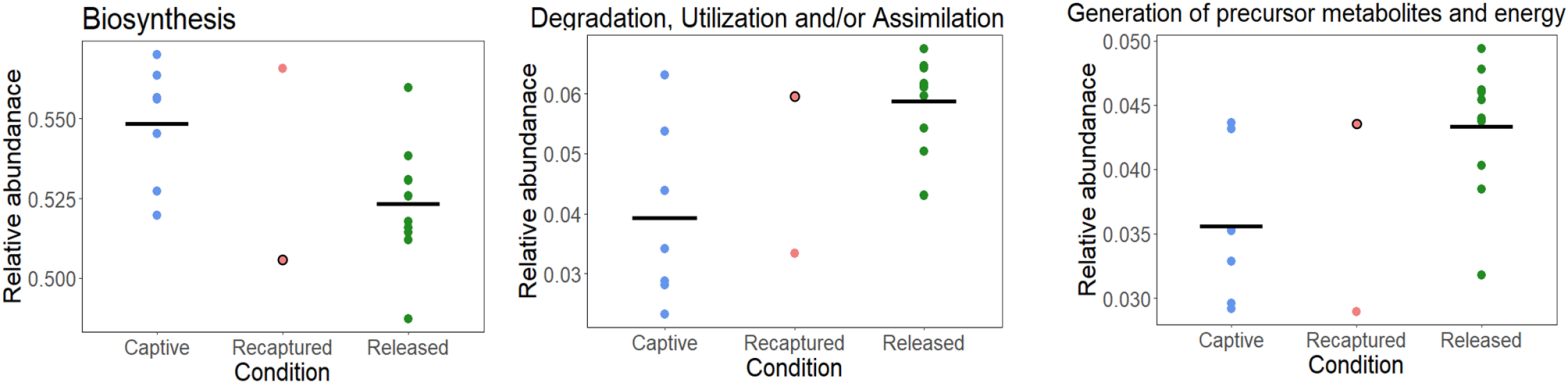
Differences in relative abundance of gut microbiota functional pathways between conditions. Samples with dark border represent the first sample collected from the individual back to captivity.

Lastly, we found that three different functional groups differ between the captive, released, and recaptured conditions. Biosynthesis associated pathways were higher in samples from the captive condition, while degradation, utilization, and assimilation pathway and generation of precursor metabolites and energy associated pathways were higher in the released condition (Fig. S8). The recaptured condition had a large variation, the first sample collected after returning from the wild appear to be more similar to samples from the released condition, while the second one collected three months later (300 days after recapture), was more similar to the ones in captivity (Fig. 6).

## Discussion

We documented for the first time, changes in the gut microbiota of primates during a reintroduction process, and, as predicted, we found that bacterial diversity was higher in samples from released individuals compared to captive ones. It is known that microbial diversity decreases according to the severity of captivity, with the highest diversity observed in primates living under the most natural conditions and the least under less natural conditions^9^. Additionally, primates show a lower bacterial alpha diversity compared to conspecifics in natural conditions^9,11^ and this reduction in bacterial diversity may be because they lose their natural microbial diversity as a consequence of diet changes since primates in the wild eat a higher diversity of plants, and captivity represents a strong change in their natural diet^10^. Another factor that may be associated with changes in gut microbiota diversity is that exposure to fecal material from other individuals and species can increase microbial diversity in nature, and in captivity this diverse inoculation no longer occurs, so individuals are exposed to fewer microorganisms from the environment^24^. In this study, individuals in captivity could constantly get reinfected by contact with feces of the other conspecifics in the same enclosure, reducing their bacterial gut diversity. Also, it has been reported that an increase in social interactions is associated with a higher diversity of gut microbiota communities^14^, but in our study we found the opposite (Table S2). The reason why we did not found a positive relation between social interactions and gut bacterial communities may be due to the fact that in captive conditions, there is a lower microbial diversity in the environment product of a homogenized diet, further suggesting that diet is a stronger factor than social interactions affecting the microbial composition.

We did not find any correlation between days after release and bacterial diversity. This is a consequence of an increase in diversity immediately after the liberation, which is then stabilized and finally, the curve decreased probably because of a regularization of suitable food and feeding sites since individuals shows a shift in diet and start consuming more food found in the forest. In addition, diet and patterns of nutrient intake can change dramatically between seasons^27,28^, and the gut microbiota is expected to adjust or adapt to the host diet^29^. In this scenario, released individuals experience a shift in diet that drive changes in the gut microbiota, and may be the reason why this condition had a higher diversity. Released individuals explore different resources (not necessarily eaten by wild primates) but keep also an alternative diet matching captivity, resulting on a mixture of bacteria from captivity, and bacteria associated with the liberation site. This is the case of macaques in the wild, that when they are given food supplies, they display a greater diversity than those in the traditional wild diet allowing them to digest new and different types of food^30^.

The weighted beta diversity analysis grouped samples by condition, and the unweighted grouped samples by the identity of the individuals, independent of the condition. This suggests that sample clustering was driven by the relative abundance of bacterial taxa but also by the presence or absence of some key taxa. Since only in the unweighted analysis samples grouped by individuals, we suggest that each primate harbor different patterns of bacteria with low abundances. This may also suggest that individuals are grouping by age in which the two adults show a higher similarity of gut microbiota composition compared to the juvenile, however, we cannot have certainty because we only have one individual per age-sex category. The differences among conditions can be explained by the fact that colonization of microbiota occurs mainly by horizontal transference by contact with the environment^31,32^ and differences in diet between the two sites could derive in differences in microbial composition. The differences between individuals may be driven by life history because infants seem to acquire microbiota from their mother and colonization continues through social interactions with conspecifics and the environment^33^. Our results also suggest that time after liberation is associated with changes in bacterial composition and the more time individuals spend in liberation the less their microbiota resembles the one in captivity.

At a phylum level, we found that the most abundant sequences correspond to phyla Firmicutes, Proteobacteria, and Bacteroidetes, which are the most abundant phyla in mice and humans^34,35^. We found that Proteobacteria is the only phylum whose relative abundance was different between conditions, being higher in captivity. This phylum is commonly found at a higher relative abundance in captive mammals^10^ and contains several pathogenic microorganisms that can alter gut microbiota^36,37^. At the genus level, we identified four bacteria that were dominant in captivity samples (*Ruminobacter*, *Campylobacter*, Unclassified S24-7, and *Sharpea*) and four in released ones (*Bacteroides*, *Sutterella*, Unclassified Coriobacteriaceae, and *Succinatimonas*). *Bacteroides* and *Sutterela* abundance were higher only in released individuals, and lower in captivity, even in the recaptured individual. The genus *Bacteroides* is associated to plant nutrient intake, mainly leaves that contain high fiber content^38^, which are more accessible to released individuals. *Sutterella* is considered a beneficial bacterium being present in healthy boars^39^ and consider as a marker of healthy gut microbiota in giant pandas and thus suitable for reintroduction processes^40^. Conversely, *Campylobacter* was exclusively found in samples from captivity, and is constantly associated to unhealthy non-human primates microbiota^41^, and in macaques is associated to periods of food scarcity^33^ suggesting that in the absence of their natural diet or periods of low abundance of diet items, animals cannot develop an optimal and healthy microbiota.

We found that the percentage of food the individuals ate from the forest was directly associated with bacterial diversity. Various studies have found that dietary niche plays an important role determining gut microbiota^2,11^, the diversity, richness, and composition in howler monkeys (*Alouatta pigra*) vary in correlation to diet, where howlers occupying suboptimal habitats consume a less diverse diet leading to less microbial diversity^20^. In our study, the released individuals explored new resources which may have contributed to an increase in their gut microbial diversity.

The condition modifies microbial composition as well as microbial function. We found that degradation, utilization and assimilation pathway, and generation of precursor metabolites and energy-associated pathways, were higher in samples from released individuals when compared to captive ones. It has been found in folivorous primate *Alouatta palliata,* a species belonging to the same family (Atelidae) as *Lagothrix spp*, that captive individuals reduce the relative abundance of metabolic pathways associated with degradation mainly because of a reduction in their native diet^9^. Interestingly, in woodrats (*Neotoma albigula*), when fed with an artificial diet, there was an increase in metabolism pathways associated with biosynthesis, and a reduction in microbial metabolism associated to degradation, specifically of secondary compounds^42^. This is the same pattern that we found in woolly monkeys, where released individuals can consume different resources with secondary compounds that cannot be found in captivity, changing the microbiota functionality. These results suggest that gut microbiota may be highly resilient to perturbations at least in a functional way, suggesting that microbiota function may be recovered when giving an appropriate diet to captive individuals.

The variation in gut microbiota found in this study was mainly associated to diet and individual, this has to be taken into account in reintroduction processes, since despite finding differences in composition, we also found differences at the level of functionality that can affect the fitness of individuals, and therefore, the reintroduction success. For example, in this study the abundance of bacteria from the phylum Proteobacteria was higher in captivity; and as we mentioned before, this genus includes several pathogenic and opportunistic microorganisms that may be harmful to the individuals. It is important then to determine at what scale changes or presence of some key bacterial taxa in gut microbiota affect the fitness of individuals that are being reintroduced, or if they are just diet-driven changes that will not affect the survival of the released individual. Knowing this will allow determining if some individuals are more suitable to be reintroduced, so we encourage deeper investigations in the gut microbiota during reintroduction processes to raise and determine better conservation strategies or at least good maintenance of individuals in captivity providing them with an appropriate diet.

## Methods

### Study sites

All the project activities were performed by Regional Governmental Institutions (Corporación Autónoma del Alto Magdalena, Cormacarena, and some others) in collaboration with Universidad de los Andes. The project have followed the ARRIVE guidelines^43^ and have the legal and ethical permits including in Colombia decree 1376 of June 27, 3013 that states “Permit to collect specimens of wild species of biological diversity for non-commercial scientific research” and the approval from Universidad de los Andes ethic committee.

The site where primates were in captivity was the Center of Attention and Valuation of Wild Fauna (Centro de Atención y Valoración de Fauna Silvestre, CAV for its acronym in Spanish) located at Teruel, Huila (2° 49′ 53.93″ N, 75° 50′ 0.775″ O). This site is located at an altitude of 910 m.a.s.l. with a mean annual precipitation of 1635 mm. Temperature ranges from 19.1 to 30.3 °C.

The site for release was located at El Pensil, Huila (1° 45′ 43.949″ N, 76° 17′ 11.68″ O), at a distance of 129 km from the captive site. This liberation site was selected for the reintroduction process because it is a Biological Corridor connecting two National Parks (Cueva de los Guacharos and Puracé) that have reported viable populations of woolly monkeys^44^. It is located at a mean altitude of 1850 m.a.s.l. with a mean annual precipitation of 2284 mm. Temperature ranges from 12 to 20 °C. It has a forest matrix consisting primarily of secondary and primary forest. This liberation site shows high abundance of plant species that have been reported to be important in the diet of wild woolly monkeys as are plants belonging to the genus *Hedyosmum sp, Miconia sp, Nectandra sp, Saurauia sp, Vismia sp, Clusia sp, Tapirira sp, Guatteria sp, Cecropia sp and Ficus sp*.^44,45^.

### Study subjects

The three individuals chosen for this study were captured as infants from their native habitat for illegal trafficking and had always lived in captivity. The group consisted of one adult male (Hodor), one adult female (Yara), and one juvenile female (Arya). For the taxonomic classification of each individual, we consider the geographic ditribution of the species based on Botero et al. (2010)^46^ and Di Fiore et al. (2014)^47^ that states that there are only two subspecies of woolly monkeys in Colombia, *Lagothrix lagothricha lugens* and *Lagothrix lagothricha lagothricha*. Also, we used characteristics of the coat color based on the descriptions made by Fooden (1993)^48^. The adult male and the adult female shows a gray to blackish color, so we assign them to *L.l. lugens* subspecies. The juvenile female shows a brown hair, so we identify her as *L.l. lagothricha.* However, this identification is not entirely precise since these subspecies in Colombia shows high gene flow between them carrying with inconsistencies in subspecies differentiation based on coat color^49^. Individuals shared a 260 m^3^ enclosure with other 10 individuals. We chose our three study subjects based on various criteria. First, the resemblance of their behavior to individuals of wild populations^50^, meaning they had similar feeding, resting, and moving patterns (Table S2). Second, whether the individuals consistently used the upper area in the enclosure because wild woolly monkeys rarely go down to the ground and by using upper strata, they reduce the risk of predation in the liberation site. Third, that individuals showed affiliative social interactions that will suggest that they will not disperse from the group after the liberation. The last parameter was that individuals were not too attached to people (caretakers and researchers) but neither too aggressive so that the monitoring could be done. If individuals met most of these criteria, they were considered good candidates for the process of rehabilitation and release.

### Behavioral data and fecal sample collection

We collected behavioral data and fecal samples as part of a larger study from May 2017 to June 2018. Data collection in captivity started in May 2017 until the released date in August 2017. Data collection for the released individuals was taken since the liberation day until March 2018. The adult female, however, was recaptured one month after the liberation because she continued to disperse away from the other two released individuals. After recapture, the adult female was returned to captivity and we continued collecting behavioral data in the enclosure using the methods previously described.

We conducted focal-animal sampling for five days per month^51^. We started recording data at 06:00 h until 18:00 h, recording each individual activity (moving, feeding, resting, or engaging in social interactions) every ten minutes. When the individual was feeding, we recorded the item (fruit, leaves, arthropods, or other) and if it was a food item native to the forest, or whether it was provisioned as part of the supplementary diet (Table S3). Social interactions were determined by the percentage of times that an individual was in direct contact with another individual.

Between May of 2017 and June 2018, we collected six, eight, and five samples from the juvenile female, adult male, and adult females, respectively, for a total of 19 samples to be assessed for microbiota composition analysis (Table S4). All fecal samples were collected immediately after defecation to avoid environmental contamination. With the help of sterile wooden sticks, approximately 2 g of feces with no direct contact to the ground were collected in a 2 ml sterile Eppendorf containing 1 ml of RNA later. Samples were homogenized by shaking and the individual identity, study site condition, and date, were recorded. Samples were stored at room temperature until the transportation to Universidad de los Andes, Colombia, where samples were stored at − 20 °C until further analysis.

### Primates diet

The individuals in captivity were fed twice a day with a standard diet consisting of a mixture of fruits and vegetables, which does not correspond to their diet in natural habitats (Table S5). The daily amount of food provisioned to primates (1473 g per individual) was calculated as the 20% of the average weight of each individual in the enclosure (Table S5). Two times a week, each individual was given an egg and 225 ml of liquid Pediasure®, a commercially distributed nutritional supplement for human infants, which was administrated orally with the help of a syringe. Individuals caught and ate arthropods that enter the enclosure opportunistically. Since the moment of liberation, we developed a basket system in the canopy to provision the individuals with an alternative diet. Three plastic baskets (52 × 35 × 20 cm) were placed randomly in the liberation zone at 10 m height. The food items we put in the baskets consists of a mixture of the same type of fruits and vegetables as were given to the individuals in captivity. This provisioned food was reduced over time as individuals were exploring the plants and feeding from the forest. Since the liberation, individuals received supplementary diet each day, after two weeks, the supplementary diet was reduced to every other day and after three months only 100 g of dry kibble for dogs was given to the individuals.

### DNA processing and sequencing

DNA was extracted from each sample using QIAamp DNA Stool Mini Kit (QIAGEN) according to the manufacturer instructions. PCR, library preparation, and sequencing were performed by the Molecular Biology Laboratory at Universidad del Bosque, Bogotá, Colombia. In brief, the V4 hypervariable region of the *16S rRNA* gene was amplified using the 515F/806R primers following the protocol by Caporaso et al^52^. This region was selected as it has been proven to provide a good taxonomical assignment of gut bacteria communities^53–55^. PCR products were barcoded and pooled in equimolar concentrations, determined using a Qubit 2.0 fluorometer (Invitrogen). Next, purification of the pool was performed with AMPure XP beads and pair-end sequenced using the V2 chemistry and 2 × 250 reads on an Illumina MiSeq. The resulting DNA sequences were deposited in the European Nucleotide Archive (ENA) under the study accession number PRJEB39493.

### Quality filtering

For each of the pair-end reads from captive and released condition, we removed barcodes and primers with cutadapt v.1.12^56^. Sequence reads were checked for quality using fastqc^57^ to establish an optimum quality threshold for the filtering step. We performed a length and quality trimming using Trimmomatic v.0.36^58^ with a sliding window of 4 bp, a quality threshold of 20, and a minimum fragment length of 55. We then used FLASH v. 1.2.11 to merge paired-end reads with default parameters except for minimum overlap where 200 bp was used^59^. Lastly, we performed another trimming process of the merged sequences using the same parameters mentioned above.

### Data processing and analysis

To process sequences between captive and released conditions we used QIIME2^60^ platform. Dereplication and denoising were performed by deblur^61^ using a truncation length of 240 bp. We next filtered the singletons and ASVs with a minimum abundance of 5% in all samples and rarefied at 13 134 sequences per sample, being the number of clean sequences for the sample with the minimum number of reads. Lastly, we performed the taxonomic assignment by training a Naïve Bayesian classifier with the Green Genes database as a References^62^. We used the same database to generate a phylogenetic tree by the method of insertion placement. To determine functional diversity, we used the PICRUSt2^63^ algorithm searching for pathway abundances between the different conditions.

The ASVs table was exported to R where we used the phyloseq package v. 1.24.2^64^ and determined alpha diversity (Shannon index) and beta diversity (weighted and unweighted UniFrac distances^65^). We conducted an Adonis test to determine statistical differences in beta diversity analysis. We ran linear mixed models in R (lme4 package v. 1.1–23^66^^)^ to assess the influence of time after liberation and diet consumed collected with animal focal-method (i.e., percentage of forest vegetables, percentage of forest fruits, percentage of arthropods, percentage of other food items, and overall percentage of forest diet (fruits and vegetables combined)), on alpha diversity of bacteria (Shannon and Chao 1 index), using the identity of each individual as a random effect variable. To evaluate the diet consumed by the individuals, we used a model selection approach based on the Akaike information criterion (MuMIn v. 1.43.17^67^), selecting the model with the lower AIC score and the higher weight^68^. To determine differences of bacterial communities over time, we performed a beta group significance analysis. This analysis will test if the distance between samples within a group is more similar to each other compared to the samples in between groups. We separate samples in five categories based on the variation in bacterial Shannon diversity index over time (Fig. S1): (1) between − 100 and − 52 days prior to liberation, (2) between − 52 and 0 days prior to liberation, (3) between 0 and 100 days after liberation, (4) beyond 100 days of liberation until the end of the study for the released individuals, (5) beyond 100 days until the end of the study for the recaptured individual. These categories were used to determine differences in Shannon diet diversity over time. To visualize the abundance of the bacterial community we built heat maps between the different conditions. To reduce the dimensions of the heatmap, we decided to use number of phyla and genera with a *p*-value lower than 0.1 between conditions. Differential abundance between conditions was computed using STAMP with a Benjamini–Hochberg correction.

## Supplementary Information

Supplementary Information
