# Supplementary material for: Monitoring the variation in the gut microbiota of captive woolly monkeys related to changes in diet during a reintroduction process
Source: Sci Rep. 2021 Mar 22;11:6522. doi: 10.1038/s41598-021-85990-0 (PMC7985493; doi:10.1038/s41598-021-85990-0)
Supplement: Supplementary file 1 — Supplementary Information [file 41598_2021_85990_MOESM1_ESM.docx]

**Supplementary material**

**Monitoring the variation in the gut microbiota of captive woolly monkeys related to changes in diet during a reintroduction process**

Camilo Quiroga-González^1*^, Luis A. Chica Cardenas^2^, Mónica Ramírez^1^, Alejandro Reyes^2,3^, Camila González^4^ & Pablo R. Stevenson^1^

**Table S1.** Results of the analysis for Akaike information criterion describing Shannon bacterial diversity. The model includes all items the individuals consume from de forest (FD), fruits that individuals consume from the forest (FF), vegetables that individuals consume from the forest (VF), other items the individuals consume (O) and arthropods (A). In bold is the model with the lowest AIC value and the higher weight.

| Predictive variables | logLik | AICc | delta | weight |
| --- | --- | --- | --- | --- |
| FD | **-10,220** | **28,040** | **0,000** | **0,262** |
| FD + FF | -8,663 | 28,184 | 0,144 | 0,244 |
| FD + VF | -9,701 | 30,259 | 2,219 | 0,086 |
| FD + A | -9,755 | 30,367 | 2,327 | 0,082 |
| FD + O | -10,178 | 31,213 | 3,173 | 0,054 |
| FD + FF + VF | -8,347 | 31,310 | 3,270 | 0,051 |
| FD + A FF | -8,407 | 31,429 | 3,389 | 0,048 |
| FD + FF + O | -8,553 | 31,721 | 3,681 | 0,042 |
| FD + A + VF | -8,814 | 32,244 | 4,204 | 0,032 |
| FD + O + VF | -9,465 | 33,546 | 5,506 | 0,017 |
| FD + A +O | -9,754 | 34,123 | 6,084 | 0,013 |
| FD + A +FF + VF | -7,814 | 34,627 | 6,588 | 0,010 |
| FD + FF +O + VF | -8,037 | 35,074 | 7,034 | 0,008 |
| FD + A + FF + O | -8,367 | 35,733 | 7,694 | 0,006 |
| VF | -14,187 | 35,975 | 7,935 | 0,005 |
| A | -14,231 | 36,062 | 8,022 | 0,005 |
| O | -14,315 | 36,230 | 8,191 | 0,004 |
| FF | -14,376 | 36,352 | 8,312 | 0,004 |
| FD + A + O + VF | -8,706 | 36,411 | 8,372 | 0,004 |
| A + VF | -14,090 | 39,037 | 10,997 | 0,001 |
| O + VF | -14,172 | 39,201 | 11,161 | 0,001 |
| FF + VF | -14,175 | 39,207 | 11,167 | 0,001 |
| A + O | -14,205 | 39,266 | 11,227 | 0,001 |
| A + FF | -14,211 | 39,279 | 11,239 | 0,001 |
| FD + A + FF + O + VF | -7,630 | 39,441 | 11,401 | 0,001 |
| FF + O | -14,314 | 39,485 | 11,445 | 0,001 |
| A + O + VF | -14,086 | 42,788 | 14,748 | 0,000 |
| A + FF + VF | -14,089 | 42,794 | 14,754 | 0,000 |
| FF + O + VF | -14,160 | 42,935 | 14,895 | 0,000 |
| A + FF + O | -14,191 | 42,997 | 14,957 | 0,000 |
| A + FF + O + VF | -14,086 | 47,171 | 19,132 | 0,000 |

**Table S2.** Percentage of behavioral data of animal-focal records of each individual activity (moving, feeding, resting, or engaging in social interactions) before and after the released.

*Wild individuals data were obtained from Stevenson (2006) reports.

|  | Arya | | Hodor | | Yara | | *Wild Individuals |
| --- | --- | --- | --- | --- | --- | --- | --- |
|  | Captive | Released | Captive | Released | Captive | Released |  |
| Moving | 27 | 16 | 25 | 16 | 44 | 62 | 26 |
| Resting | 35 | 41 | 43 | 53 | 32 | 13 | 35 |
| Feeding | 30 | 40 | 27 | 29 | 20 | 25 | 36 |
| Social Interaction | 8 | 3 | 5 | 2 | 4 | 0 | 3 |

**Table S3.** Diet category and diversity of food items observed to be consumed by primates during data collection in captive and released conditions.

| Condition | Diet Category | Diet Item |
| --- | --- | --- |
| Captive | Arthropods | Flies, ants |
|  | Fruits | Banana, papaya, strawberry, cantaloupe, cucumber, grapes, guava, apple, pear, beans and corn |
|  | Other | Egg, Nutritional supplement (Pediasure®) and dry kibble for dogs |
|  | Vegetables | Lettuce, Chard and Carrot |
| Released | Arthropods | Dragonfly, Flies, ants, insect cocoons and Unclassified Arthropods |
|  | Fruits | Banana, Cantaloupe, Cucumber, Grapes, *Guettarda sp., Ficus sp., Saurauia sp., Hedyosmum sp. and* Unclassified plant fruits |
|  | Other | Egg, Nutritional supplement (Pediasure®) and dry kibble for dogs |
|  | Vegetables | Ferns, Orchid bulbs, Ericaceae leaves and flowers, Araceae leaves and inflorescence, *Besleria* *sp.* leaves, Melastomataceae leaves, *Monstera sp* leaves, *Crotton sp.* Inflorescence, Grass, Bromelia Petioles, *Selaginella sp.,* Araceae roots, *Heliocarpus sp.* shoots, Unclassified plant leaves, Unclassified plant fruits, Chard and Carrots |

**Table S4.** Data associated with the samples collected for microbiota analysis of the three studied individuals, A correspond to samples from Arya, H to Hodor and Y to Yara.

| Sample Code | Sex | Age | Individual | Date | Condition |
| --- | --- | --- | --- | --- | --- |
| A1 | F | J | Arya | 16/05/2017 | Captive |
| A2 | F | J | Arya | 12/06/2017 | Captive |
| A3 | F | J | Arya | 16/08/2017 | Released |
| A4 | F | J | Arya | 28/08/2017 | Released |
| A5 | F | J | Arya | 1/09/2017 | Released |
| A6 | F | J | Arya | 5/09/2017 | Released |
| H1 | M | A | Hodor | 16/05/2017 | Captive |
| H2 | M | A | Hodor | 12/06/2017 | Captive |
| H3 | M | A | Hodor | 12/07/2017 | Captive |
| H4 | M | A | Hodor | 17/08/2017 | Released |
| H5 | M | A | Hodor | 2/09/2017 | Released |
| H6 | M | A | Hodor | 13/11/2017 | Released |
| H7 | M | A | Hodor | 27/02/2018 | Released |
| H8 | M | A | Hodor | 11/03/2018 | Released |
| Y1 | F | A | Yara | 16/05/2017 | Captive |
| Y2 | F | A | Yara | 11/07/2017 | Captive |
| Y3 | F | A | Yara | 17/08/2017 | Released |
| Y4 | F | A | Yara | 6/03/2018 | Recaptured |
| Y5 | F | A | Yara | 11/06/2018 | Recaptured |

**Table S5.** Individual primate supplemented diet. The primates are captivity were fed twice a day, at the morning and at noon with a standard diet consisting of a mixture of fruits and vegetables, which does not correspond to their diet in natural habitats.

| Diet Item | Morning food (gr) | Noon food (gr) | | Total per day (gr) | | Type of item |
| --- | --- | --- | --- | --- | --- | --- |
| Banana | 258,4 | 77,5 | 335,9 | | Fruit | |
| Cucumber | 4,87 | 30,4 | 35,27 | | Fruit | |
| Corn | 117,7 | 0 | 117,7 | | Fruit | |
| Carrot | 114 | 34,22 | 148,22 | | Vegetable | |
| Papaya | 71,2 | 21,4 | 92,6 | | Fruit | |
| Cantaloupe | 41,4 | 12,4 | 53,8 | | Fruit | |
| Beans | 48,63 | 14,6 | 63,23 | | Vegetable | |
| Guava | 38,8 | 11,6 | 50,4 | | Fruit | |
| Apple | 27,2 | 8,17 | 35,37 | | Fruit | |
| Pear | 24,5 | 7,32 | 31,82 | | Fruit | |
| Chard | 43,3 | 13 | 56,3 | | Vegetable | |
| Grapes | 75 | 75 | 150 | | Fruit | |
| Dry kibble for dogs | 100 | 0 | 100 | | Other | |
| Strawberry | 75 | 75 | 150 | | Fruit | |
| Lettuce | 40 | 13 | 53 | | Vegetable | |
| Total | 1080 | 393,61 | 1473,61 | |  | |

**Figure S1.** Changes in bacterial Shannon and Chao1 diversity indexes as a function of time. Each individual sample is plotted at its corresponding time of collection as compared to the date of release and colored according to the individual or origin. Circled points correspond to samples from Yara after the individual returned to captivity. Dashed red line corresponds to the average of the three individuals.


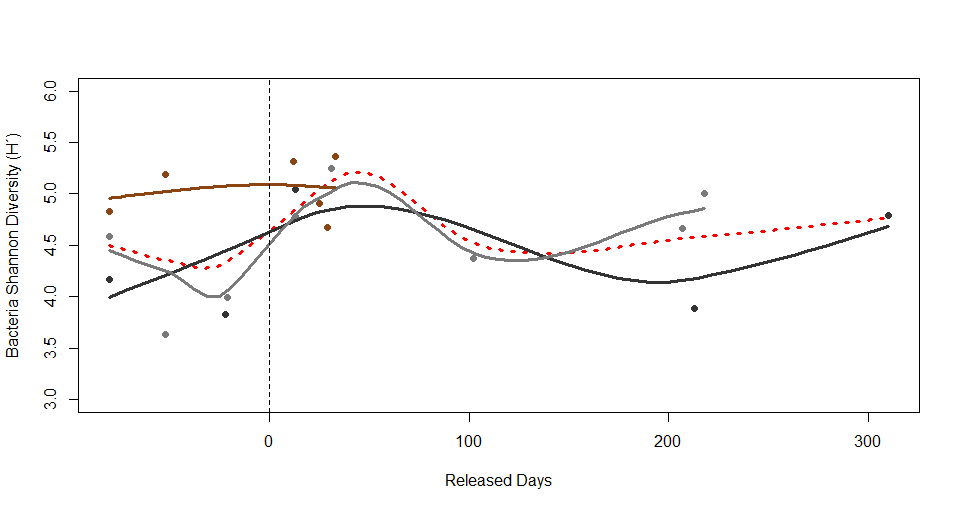

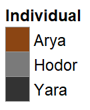

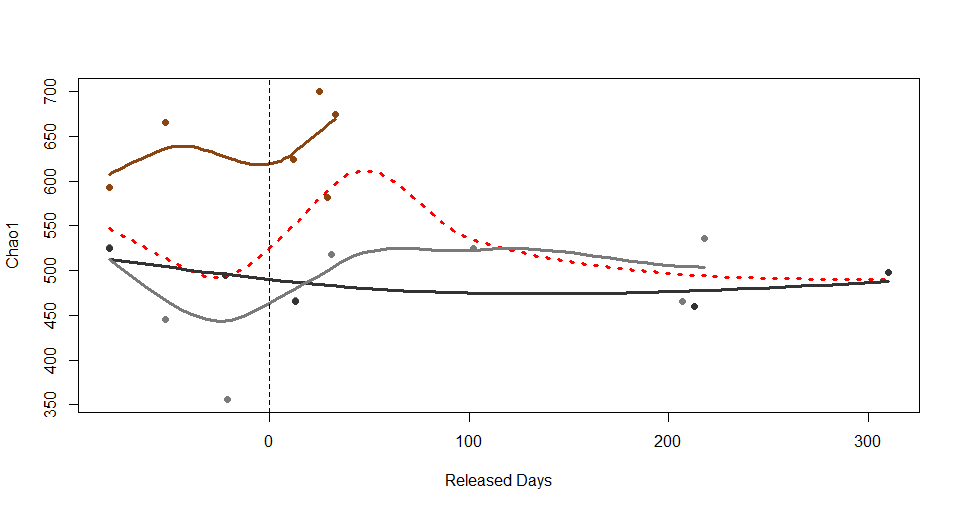

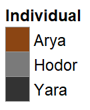


**Figure S2.** Linear mixed model of bacterial alpha diversity (Shannon and Chao 1 Index) explained by (i) the number of days after releasing (Left) and (ii) the percentage of food primates obtained from the forest (Right)


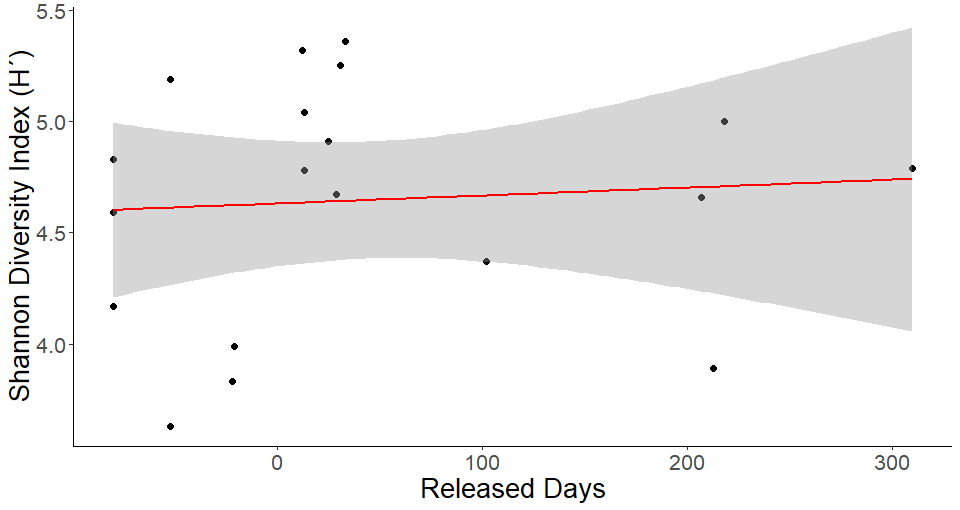

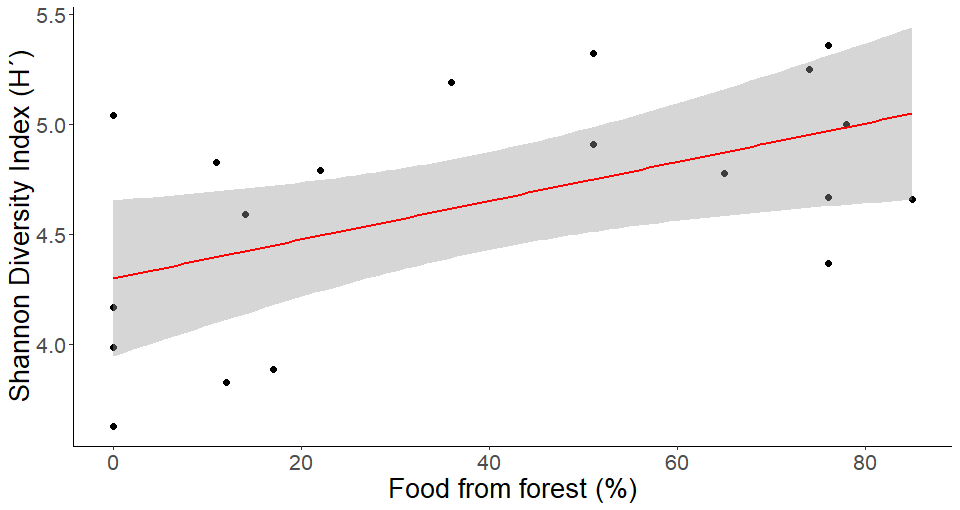

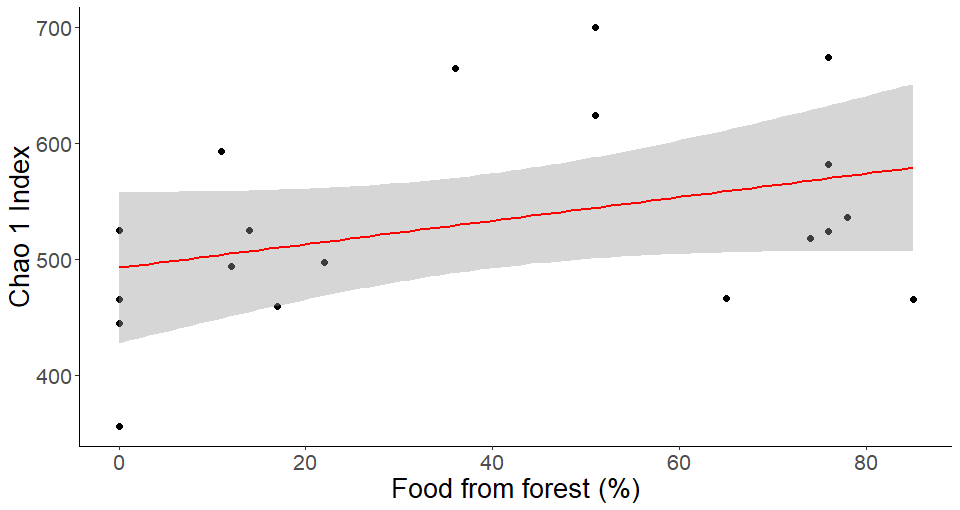

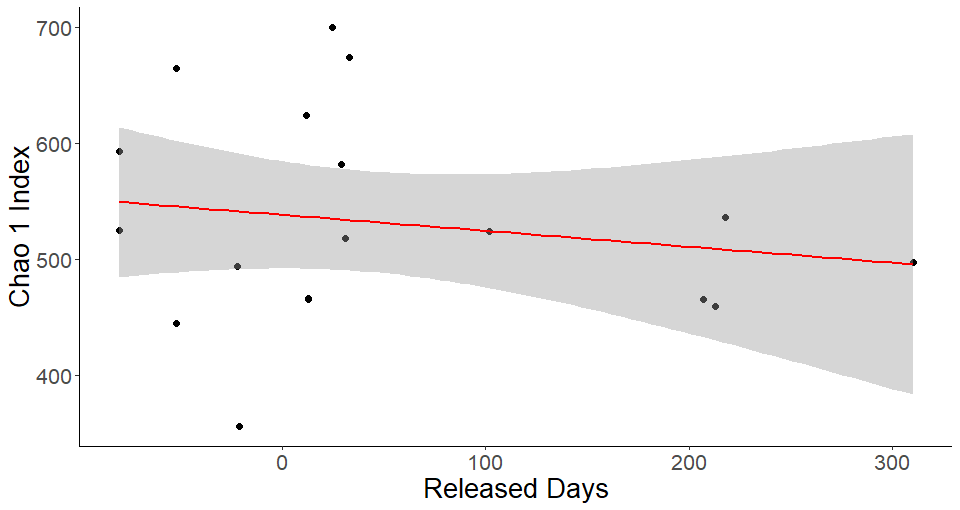


**Figure S3.** (a) Diet diversity for the different conditions of the individuals, Captive (n=9) and Released (n=10). Samples from the recaptured individual are considered as captive condition in this analysis. Shannon diet diversity was significantly different between captive and released conditions (ANOVA, *F*_1,17_ = 14.86, p < 0.001). (b) Diet diversity changes associated to the different time groups. Group 1 (n=3) : Between -100 to -52 days prior to liberation, Group 2 (n=4): Between -52 to 0 days prior to liberation, Group 3 (n=7): Between 0 – 100 after the liberation, Group 4 (n=3): From 100 days until the end of the study for the released individuals and Group 5 (n=2): From 100 days until the end of the study for the recaptured individual. Color code represents time groups of samples taken in captivity and liberation site.


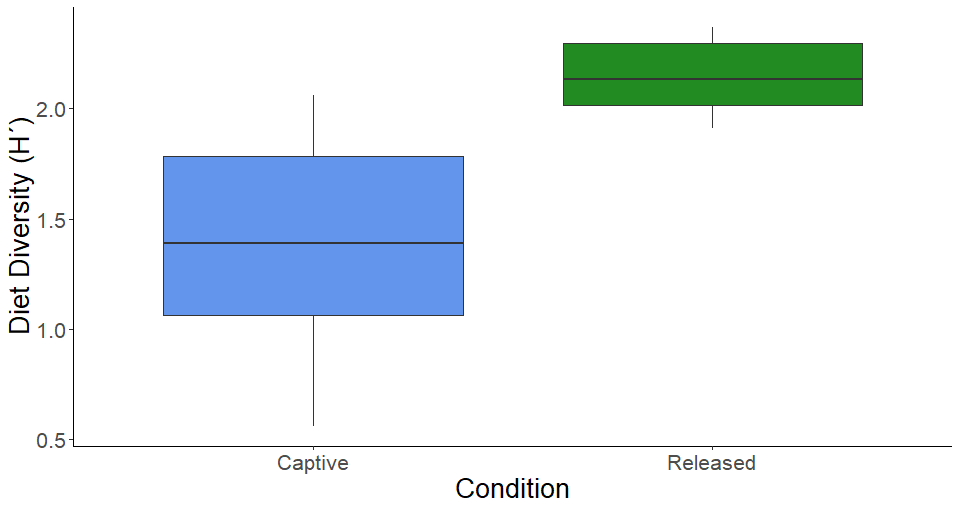

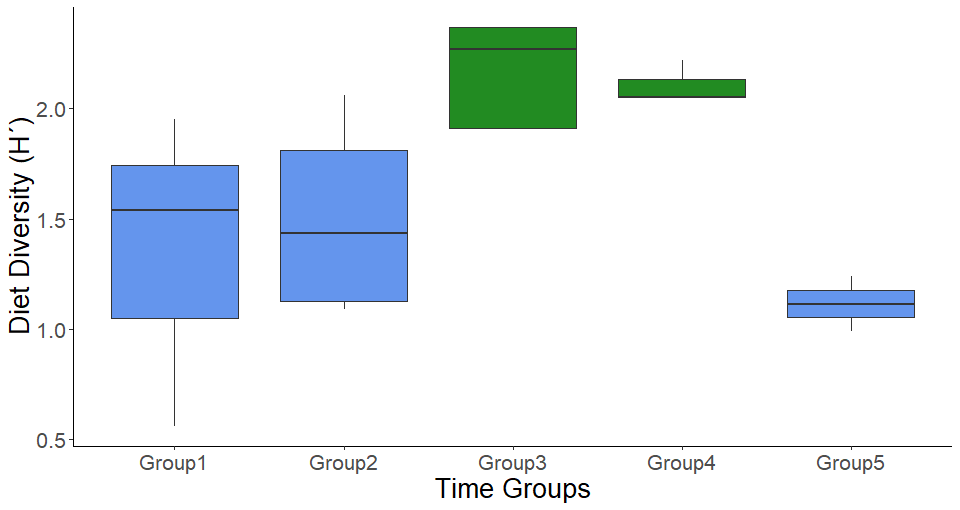

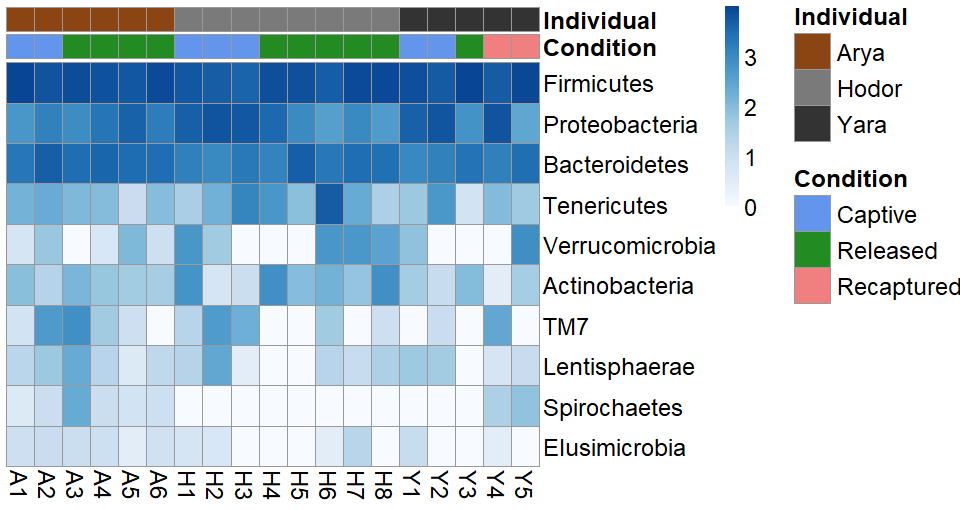


**a**

**b**


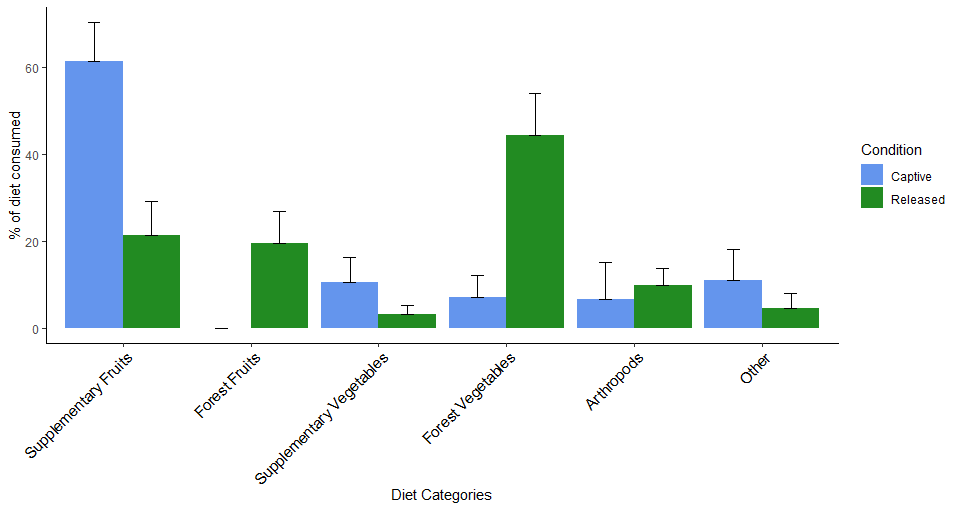
**Figure S4.** Data of the percentage of diet categories the individuals consumed during captivity and after released.

**Figure S5.** Weighted UniFrac distances of *16S rRNA* gene amplicons data grouping by condition and name of the individuals. Samples with a black border represent samples from an individual upon return to captivity after one month in the wild. Arrows follow the trajectory of samples of each individual over time.


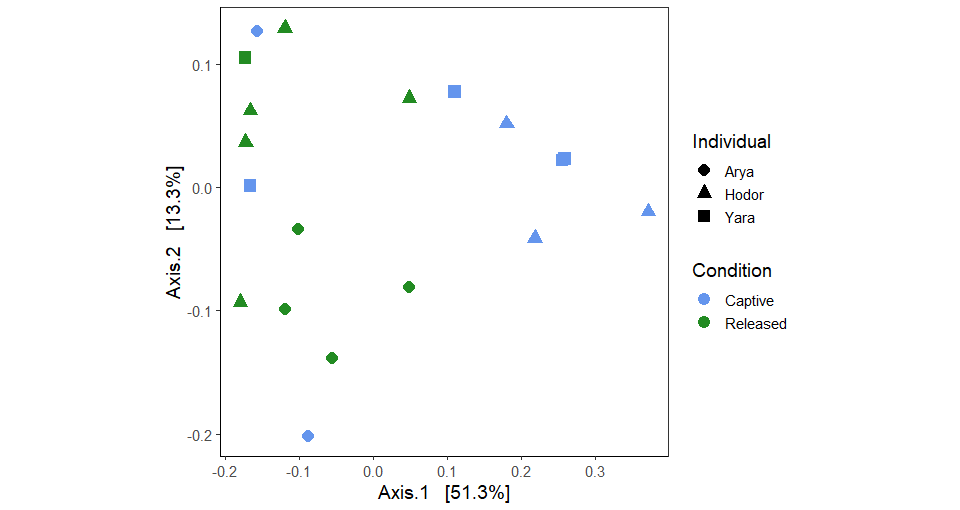


**Figure S6.** Beta group significance analysis of Weighted distances between five categories based on the time period during the reintroduction process. Group 1 (n=3): Between -100 to -52 days prior to liberation, Group 2 (n=4): Between -52 to 0 days prior to liberation, Group 3 (n=7): Between 0 – 100 after the liberation, Group 4 (n=3) : From 100 days until the end of the study for the released individuals and Group 5 (n=2): From 100 days until the end of the study for the recaptured individual. Asterisk represent statistical differences (p < 0.05) between the different groups and the group being compared.


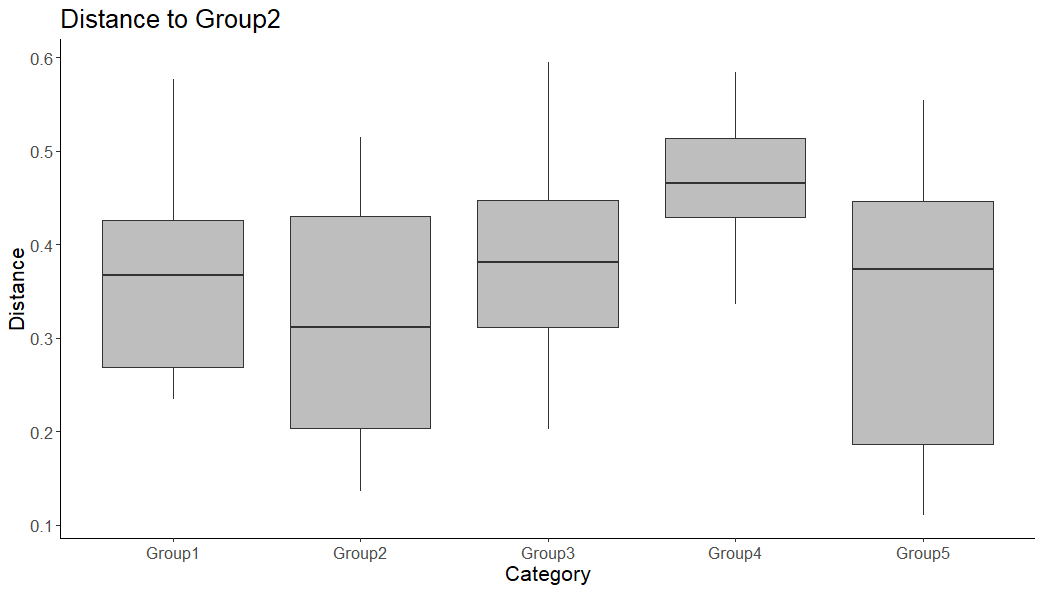

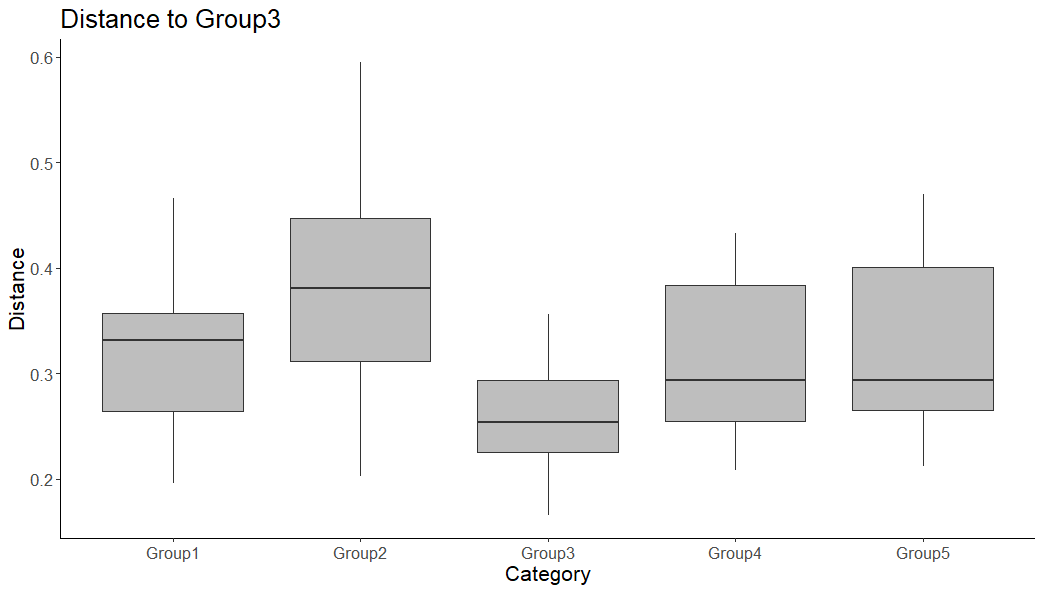


_*_


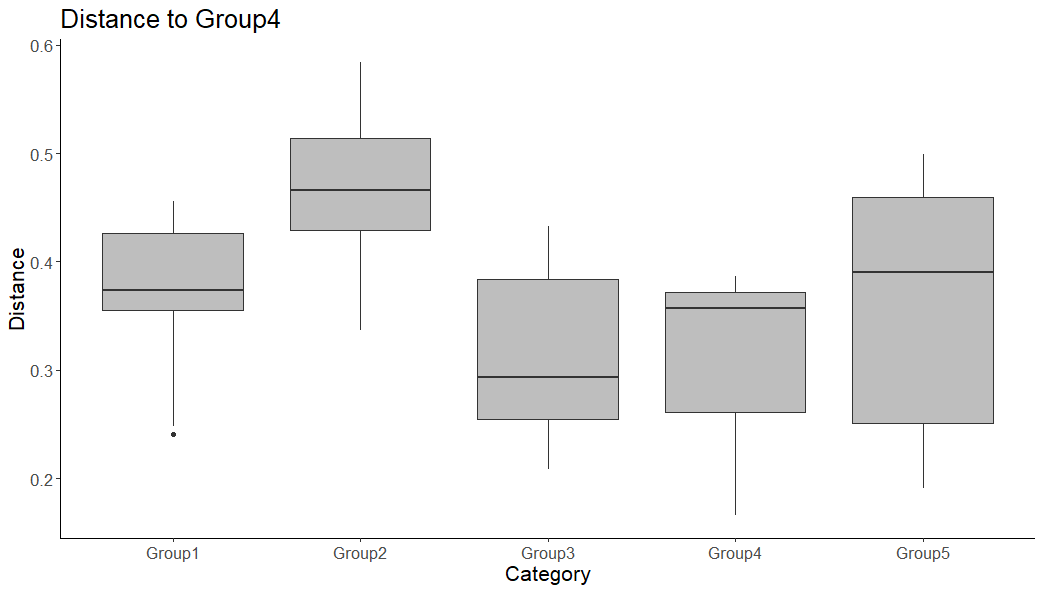


_*_


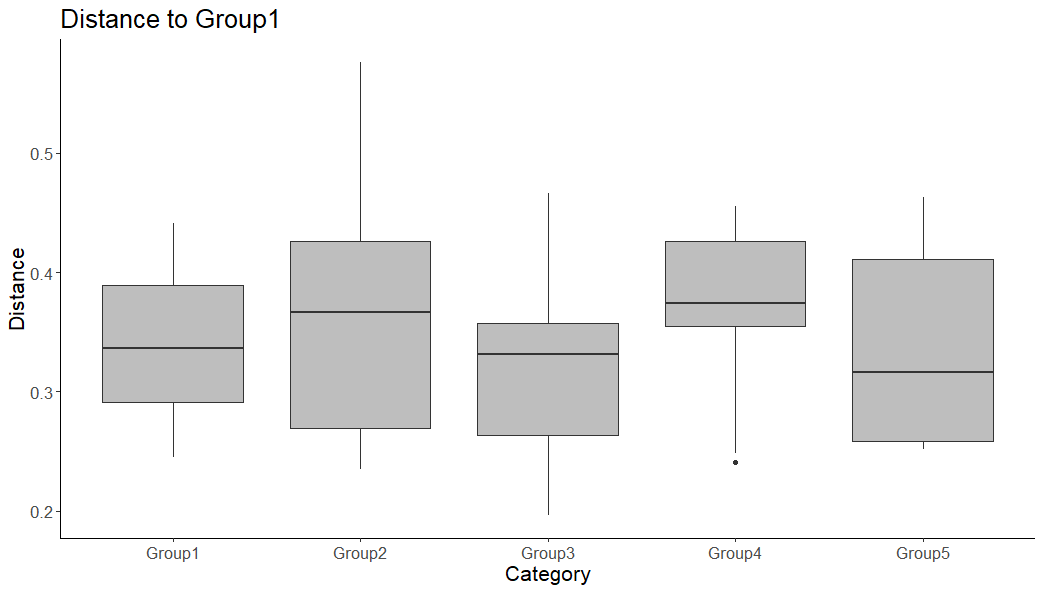

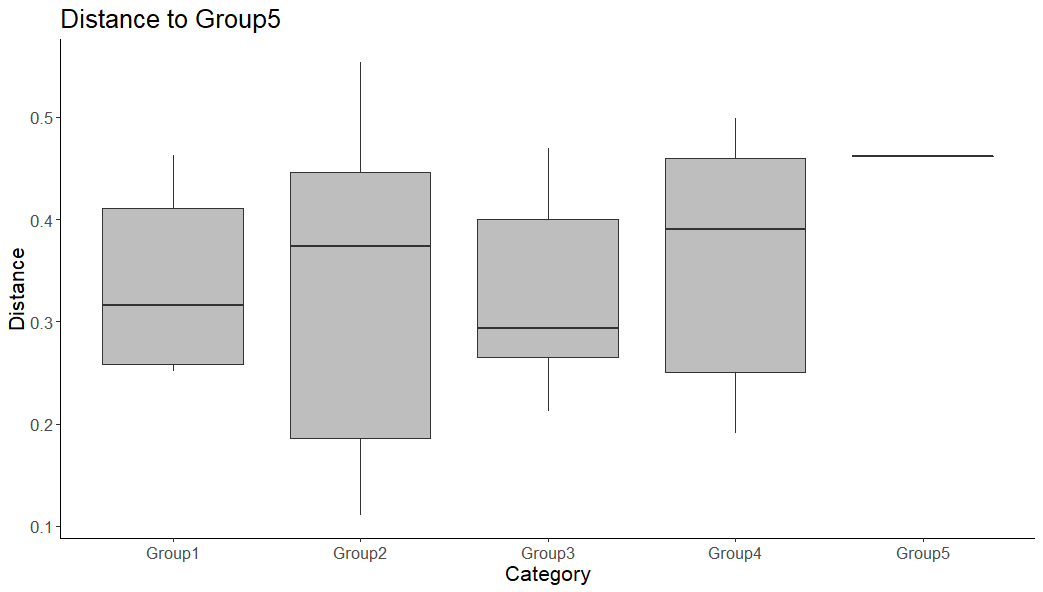


_*_

**Figure S7.** (a) Heatmap showing the logarithm of the abundance of bacterial genera found in fecal samples from captive, released and recapture individuals. Samples are sorted by individual, and within individual by time. Colored boxes on top of the heatmap indicate the condition of the sample and groups by the individual sampled. (b) Extended error bar plot with a 95% confidence interval organized by the lowest p-value showing the differences in mean proportions between captive and released individual`s samples and its associated corrected p-value (Welch’s t-test).


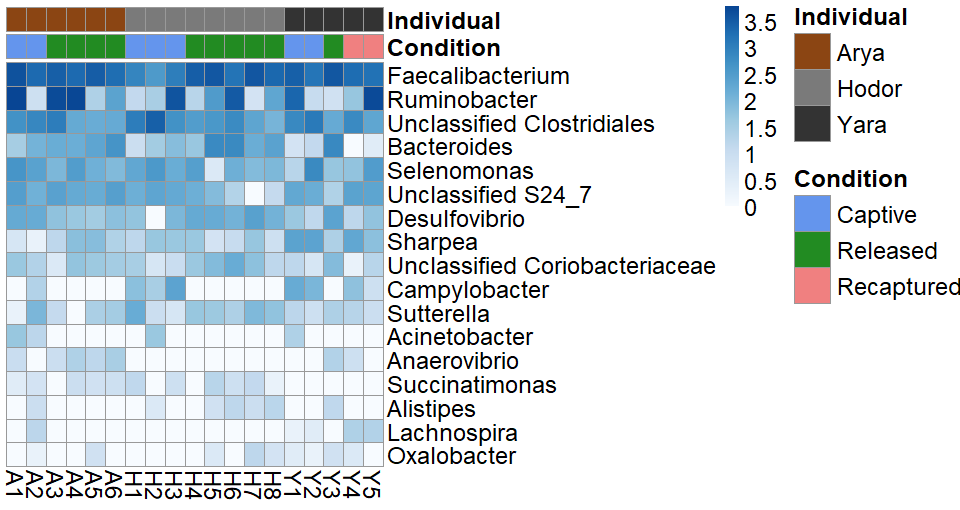

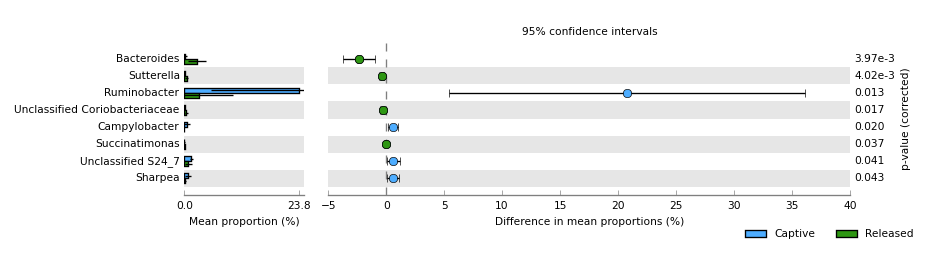


**a**

**b**

**Figure S8.** Extended error bar plot with a 95% confidence interval showing the differences in mean proportions between captive and released condition of functional diversity analysis and its associated corrected p-value (ANOVA). Samples from recapture were omitted in this analysis.

**
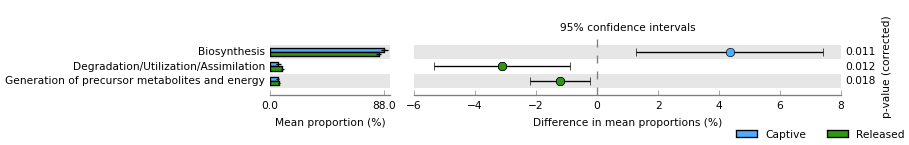
**
